# CYPminer: an automated cytochrome P450 identification, classification, and data analysis tool for genome data sets across kingdoms

**DOI:** 10.1186/s12859-020-3473-2

**Published:** 2020-04-29

**Authors:** Ohgew Kweon, Seong-Jae Kim, Jae Hyun Kim, Seong Won Nho, Dongryeoul Bae, Jungwhan Chon, Mark Hart, Dong-Heon Baek, Young-Chang Kim, Wenjun Wang, Sung-Kwan Kim, John B. Sutherland, Carl E. Cerniglia

**Affiliations:** 1 Division of Microbiology, National Center for Toxicological Research (NCTR)/U.S. FDA, Jefferson, AR 72079 USA; 2 NCTR/U.S. FDA, Jefferson, AR 72211 USA; 3 Department of Oral Microbiology and Immunology, School of Dentistry, Dankook University, Yongin, 16889 Republic of Korea; 4 Department of Microbiology, Chungbuk National University, Cheongju, 28644 Republic of Korea; 5 Department of Business Information Systems, University of Arkansas at Little Rock, Little Rock, AR 72204 USA

**Keywords:** Cytochrome P450, CYP identification, CYP classification, Pan-CYPome, CYP co-occurrence network, Data analysis, Python, Software

## Abstract

**Background:**

Cytochrome P450 monooxygenases (termed CYPs or P450s) are hemoproteins ubiquitously found across all kingdoms, playing a central role in intracellular metabolism, especially in metabolism of drugs and xenobiotics. The explosive growth of genome sequencing brings a new set of challenges and issues for researchers, such as a systematic investigation of CYPs across all kingdoms in terms of identification, classification, and pan-CYPome analyses. Such investigation requires an automated tool that can handle an enormous amount of sequencing data in a timely manner.

**Results:**

CYPminer was developed in the Python language to facilitate rapid, comprehensive analysis of CYPs from genomes of all kingdoms. CYPminer consists of two procedures i) to generate the Genome-CYP Matrix (GCM) that lists all occurrences of CYPs across the genomes, and ii) to perform analyses and visualization of the GCM, including pan-CYPomes (pan- and core-CYPome), CYP co-occurrence networks, CYP clouds, and genome clustering data. The performance of CYPminer was evaluated with three datasets from fungal and bacterial genome sequences.

**Conclusions:**

CYPminer completes CYP analyses for large-scale genomes from all kingdoms, which allows systematic genome annotation and comparative insights for CYPs. CYPminer also can be extended and adapted easily for broader usage.

## Background

Cytochrome P450 monooxygenases (termed CYPs or P450s) are hemoproteins that are ubiquitously found in all kingdoms, including bacteria and viruses [1]. CYPs represent one of the largest protein families and catalyze a variety of reactions in cellular systems [2]. Since the first publication on “cytochrome P450” in 1962 [3], CYPs have been extensively researched in relation to drug metabolism, and genotypic and phenotypic evolution [4–6]. More than 95,000 articles have been retrieved in PubMed when searched using the keyword “cytochrome P450”.

CYPs are biological catalysts with a wide range of catalytic activities [4, 5]. Their notorious metabolic pleiotropy and epistasis are closely associated with pathogenesis, the utilization of specific substrates, the detoxification of xenobiotics and drug metabolism [4, 7, 8]. Thus, functional identification and classification of the entire CYP complement of an organism (i.e., the CYPome) is an important step to draw a biochemical and metabolic blueprint for that species. A real scale-free metabolic network can be constructed based on the blueprint and functional genomic data. Such a scale-free network provides insights into the pleiotropic and epistatic metabolic behavior of the CYPome. Several researchers have unraveled CYPomes from different organisms across kingdoms, followed by analyses of CYP diversity and evolution [6, 9, 10]. Functional and evolutionary analyses of fungal CYPs have been pivotal in understanding the ecological specialization and functional diversification of individual fungal taxa [10]. The comparative CYPome analyses of 60 mycobacterial species (i.e., pan-CYPome analyses) have recently enhanced our understanding of the molecular evolution of CYPs in terms of the dynamic nature across biological kingdoms [6].

The rapid advancement of technology has significantly reduced the costs of genome sequencing; thus more genome sequencing data have become available for research. CYP-centric genomic analyses, however, require high quality genome annotation, which can be only achieved by sustained computation and manual curation efforts. With the exception of a few reference genomes, genome annotation, however, is often incomplete. Robust and complete CYP annotation is important for a genome to be fully utilized. To do so, computational tools should be employed to handle the rapid influx of genome sequences and support large-scale comparative CYPome analyses systematically across kingdoms. Although currently web-based CYP identification and classification systems are available [10, 11], no automated program for systematic CYPome analyses at the genomic population level (a collection of genomes) has been introduced. We, therefore, have developed a software called CYPminer, which was designed to facilitate rapid, comprehensive genome annotation and comparative analyses of CYPs from all kingdoms.

## Implementation

CYPminer is written in Python 2.7 and packaged as an executable file for Windows. The program requires two external programs called USEARCH [12] and RPSBLAST [13] and two databases (i.e., CYPdb_usearch and CYPdb_rpsblast). These programs and databases should be individually downloaded, and their locations should be provided to CYPminer. Users are able to freely download USEARCH (https://www.drive5.com/usearch/download.html), RPSBLAST (ftp://ftp.ncbi.nlm.nih.gov/blast/executables/blast+/LATEST/), and the databases (https://github.com/Okweon/CYPminer).

**Fig. 1:**
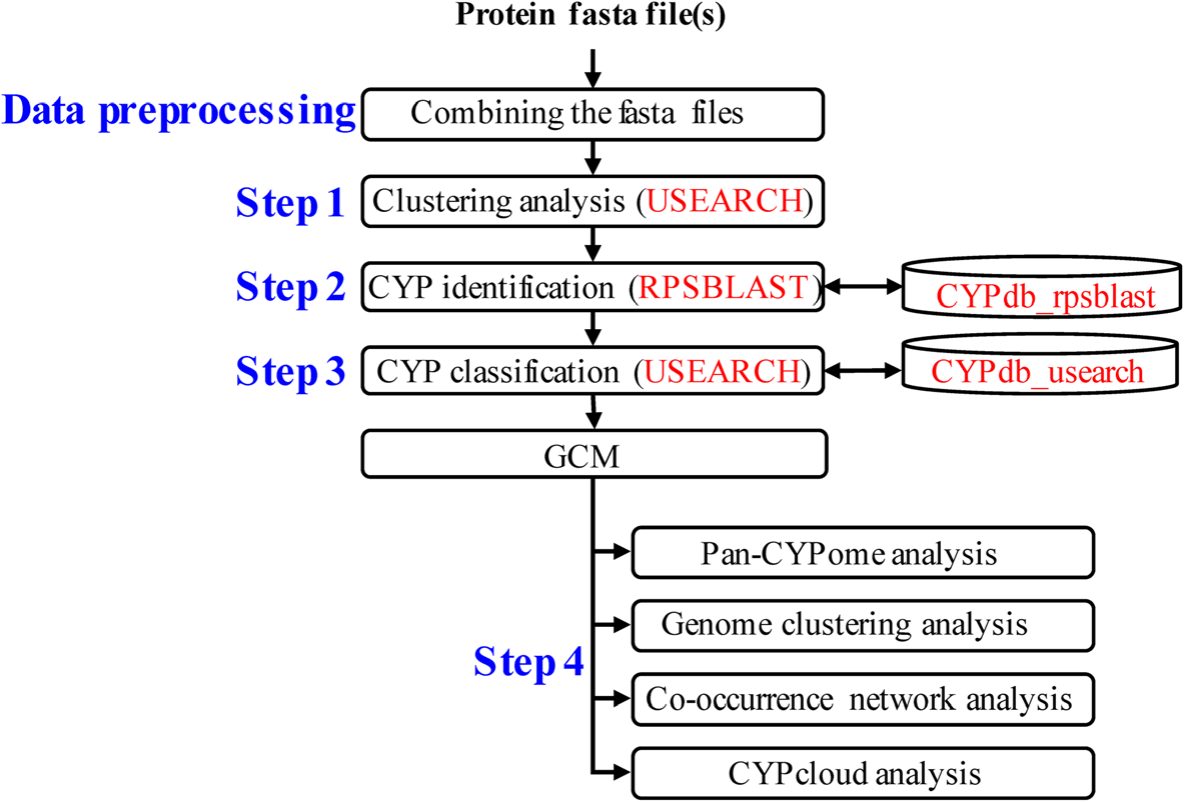
CYPminer workflow. Please refer text for details

**Fig. 2:**
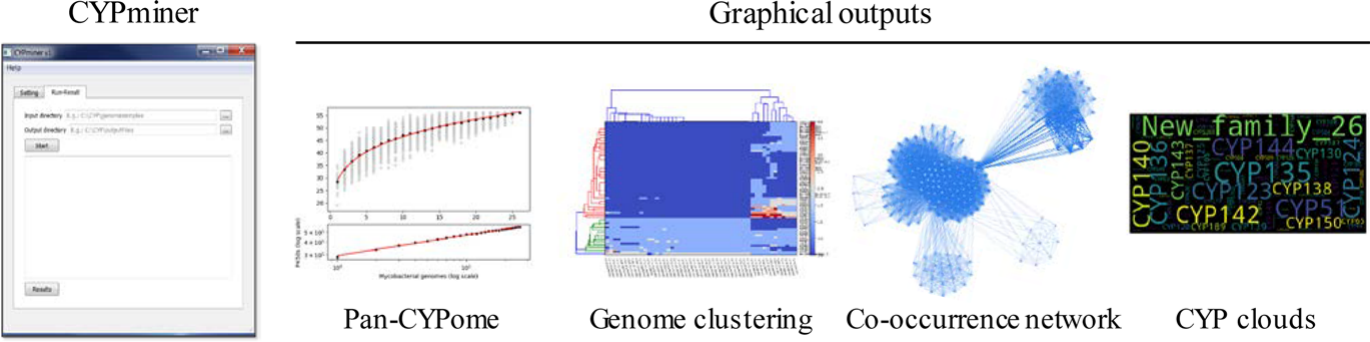
The GUI and graphical outputs of CYPminer. The CYPminer GUI has a tab pane with two pages, Setting and Run-Result, respectively. In the setting page, user can provide the paths for the two programs (USEARCH and RPSBLAST) and databases (CYPdb_usearch and CYPdb_rpsblast). In the Run-Result page, user can provide the paths for input and output directories but also see the analysis progress, allowing for easy checking at a glance. For detailed information on the graphical outputs, please see the text

The overall workflow of CYPminer is depicted in Fig. 1. CYPminer supports protein FASTA files (.fasta or .faa) as its input. In the data preprocessing step, CYPminer processes the input FASTA files for orthologous clustering analysis (Step 1). CYPminer first constructs a Genome-CYP Matrix (GCM) via the CYP identification (Step 2) and classification processes (Step 3) and then conducts its analyses and visualization outputs, such as pan-CYPomes (pan- and core-CYPome), CYP co-occurrence networks, CYPclouds, and genome clustering (Step 4). Examples of these outputs are described in Fig. 2. CYPminer was tested with diverse CYP sequences from all kingdoms.

### Step 1: orthologous clustering to select representative sequences

CYPminer adopts a clustering-based strategy to minimize redundancy in a protein sequence space to handle large genome datasets within a reasonable run time. CYPminer utilizes the UCLUST clustering algorithm in USEARCH [12]. To select a stable, scalable and unbiased representative sequence set of a genome population (a collection of genomes), the sequence identity value of 55% is employed as a cut-off for clustering. The cut-off value satisfies the requirements for systematic CYP-centric analyses and the identity value recommended for proteins (≥50%) [12]. Additional clustering options, −centroids and -uc, are used to generate a FASTA file, with cluster centroids (i.e., representative protein sequences), and a UCLUST-formatted text file, with cluster information (the output filename, R_clusters.uc), respectively. These output files are provided for CYP identification, classification, and GCM generation in the next steps.

### Step 2: identification of CYP protein(s)

The representative sequences are subjected to RPS-BLAST with a harsh expectation cut-off value (0.00001) to identify CYPs by comparing a query protein sequence against the CYP-specific conserved domains. CYPminer uses the local database called CYPdb_rpsblast, which is a sub-database containing only CYP domains, to identify sequences with CYP domains. The conserved domain searching strategy—using the representative sequences and the CYP specific local domain database—allows researchers to see ultra-fast CYP identification.

### Step 3: classification of CYP(s)

For classification, protein sequence(s) with the conserved CYP domains are retrieved and further subjected to USEARCH BLAST against the local database named CYPdb_usearch. The database was reconstructed with known CYPs at the Cytochrome P450 Homepage [11]. According to the International P450 Nomenclature Committee rule [14], proteins with ≥40% identity and ≥ 55% identity are classified under the same family and subfamily, respectively. As a result, CYPs that show less than 40% identity to known CYPs are assigned to new P450 families and subfamilies.

### Step 4: GCM analysis

After the classification process (Step 2), CYPminer parses the uc file (Step 1) and the classification output (Step 3) to generate GCMs, weighted by the frequencies of CYPs in the genomes. A GCM is basically a matrix, with CYPs designated by rows and genomes by columns, whose elements are the counts of CYPs. Subsequent analysis is based creatively on GCM. CYPminer performs four different types of GCM analyses as follows:

(i) Pan-CYPome analysis

Similar to the general pan-genome analysis [15, 16], CYPminer yields both graphical and text outputs for pan- and core-CYPome. The pan-CYPome describes the full complement of CYPs in a genome population and the core-CYPome indicates CYPs present in all individuals. To analyze the pan-CYPome profile of large-scale genomes efficiently, CYPminer randomly samples with repeats of 300 times (non-redundant combinations), and their average value ${\overset{=}{G}}_{pan}(n)$ (and ${\overset{=}{G}}_{core}(n)$) will be calculated as the pan-CYPome size and core CYPome size of *n* genomes, respectively. The pan-CYPome size (*C*_*pan*_) and core-CYPome size (*C*_*core*_) after addition of each genome is calculated based on following the formulas:

1 $$ Cpan=\sum \limits_{n=1}^n fpan(Ci),\kern0.5em fpan(Ci)=\left\{\begin{array}{c}1\kern0.5em if\  Gci\ge \kern0.47em 1\kern0.5em \\ {}0\kern0.5em if\  Gci=0\end{array}\right. $$

2 $$ Ccore=\sum \limits_{n=1}^n fcore(Ci),\kern0.29em fcore(Ci)=\left\{\begin{array}{c}1\kern0.47em if\  Gci= Gt\\ {}0\kern0.47em if\kern0.27em Gci\kern0.40em \ne \kern0.45em Gt\end{array}\right. $$

Where *C*_*i*_ represents the *i*^th^ CYP family/subfamily, *G*_*ci*_ and *G*_*t*_ represent the sizes of the genome(s) with *C*_*i*_ and the dataset, respectively, *n* is the total number of CYP families/subfamilies obtained from the entire dataset and pan/core genome size, (C_*pan*_*/C*_*core*_) represents the size of the pan/core genome after addition of the *n*^th^ genome from the dataset. CYPminer performs power-law regression by the regression function *n* = σ*N*^γ^ to model the median sizes of the pan-CYPomes, where *n* is the total number of CYPs in the pan-CYPome, *N* is the number of genomes considered, and σ and γ are free parameters.

(ii) Clustering analysis

CYPminer uses the Python heatmapcluster library (https://github.com/WarrenWeckesser/heatmapcluster) to generate a clustered heatmap with dendrograms plotted along with the heatmap. Users are able to use the two GCMs (i.e., panTable_Family_numeric.csv and panTable_Subfamily_numeric.csv) for other external programs for additional clustering analysis and customized visualization.

(iii) Co-occurrence network analysis

CYPminer recognizes CYP co-occurrence network analysis. If two CYPs exist in a genome, these two CYPs are associated with each other and form a co-occurrence relation. In a CYP co-occurrence network, nodes represent CYPs whose edges indicate relationship between CYPs. The node size and line width are weighted by CYP occurrence counts and frequency of co-occurrence, respectively. CYPminer creates co-occurrence matrices from GCMs for family and subfamily level networks: *M*_CYP_ = diagonal (reps(*M* × *M*^*T*^)), where *T* indicates the matrix transpose. The reps() function replaces all non-zero entries of the matrix products with a 1, converting the matrices from weighted to unweighted co-occurrence matrices, and the diagonal() function resets diagonal with a 0. Using these matrices, CYPminer uses the Python pyvis library (https://pyvis.readthedocs.io/en/latest/) to reconstruct and visualize undirected CYP co-occurrence networks. CYPminer generates four network-related output files—two static html files and text files for family and subfamily networks. The two html outputs allow interactive browser-based visualizations of the co-occurrence networks. The output text files could additionally be supplied to other external tools, such as gephi (https://gephi.org/) and cytoscape (https://cytoscape.org/) for further analysis and visualization.

(iv) CYP cloud analysis

CYPminer uses the Python wordcloud library (https://github.com/amueller/word_cloud) to generate a CYP cloud, which is a visual representation of CYP frequency data in a genomic population.

CYPminer generates a maximum of 32 output files. The output files could be grouped into two groups, which consist of a raw data group, with ‘R-’ in the output filename and practical table, and a figure group, with a categorical initial name, such as ‘I’ (identification), ‘C’ (classification), and ‘N’ (network). The large table datasets (i.e., GCMs) were subjected to data visualization to generate four different categories of visual contexts (i.e., pan-CYPome, co-occurrence network, clustering, and CYP cloud), which make users easily understand the significance of table data in CYPminer.

## Results and discussion

CYPminer is a Python-based program with a graphical interface, allowing users CYP identification/classification and downstream analyses from all kingdom protein sequences in a user-friendly manner. Using fungal and bacterial genome sequences downloaded from Ensembl (https://useast.ensembl.org/index.html) and PATRIC (https://www.patricbrc.org/) databases, three test datasets were prepared to demonstrate the capabilities of CYPminer across kingdoms, which include F-10, 10 fungal genomes; B-50, 50 mycobacterial genomes, and FB-60, F-10 + B-50 (Table 1). Table 1 shows a summary of the datasets and their results.

**Table 1:** Summary of the dataset and results generated by CYPminer from the three datasets

Sample	No. of Genomes	No. of Seq.	No. of clusters	No. of CYPs	CYP classification^a^		Pan-CYPome				Dominant CYPs	Running time^b^
							Pan		Core			
					Family	Subfamily	Family	Subfamily	Family	Subfamily		
F-10	10	117,916	71,023	618	175 (25)	307 (169)	175	307	2	2	CYP65, CYP509, CYP5203	3 m 59 s
B-50	50	230,489	17,623	1286	46 (5)	65 (14)	46	65	6	5	CYP135, CYP51, CYP140	3 m 52 s
FB-60	60	348,405	88,648	1904	220 (30)	372 (183)	220	372	0	0	CYP135, CYP51, CYP140	9 m 39 s

^a^Numbers in parentheses indicate new families or subfamilies within each sample

^b^Computer system: Processor, Intel® CUP E5-2650v4@ 2.20GHz (2 processors); RAM, 64.0 GB; System type, 64-bit (Windows 10)

**Fig. 3:**
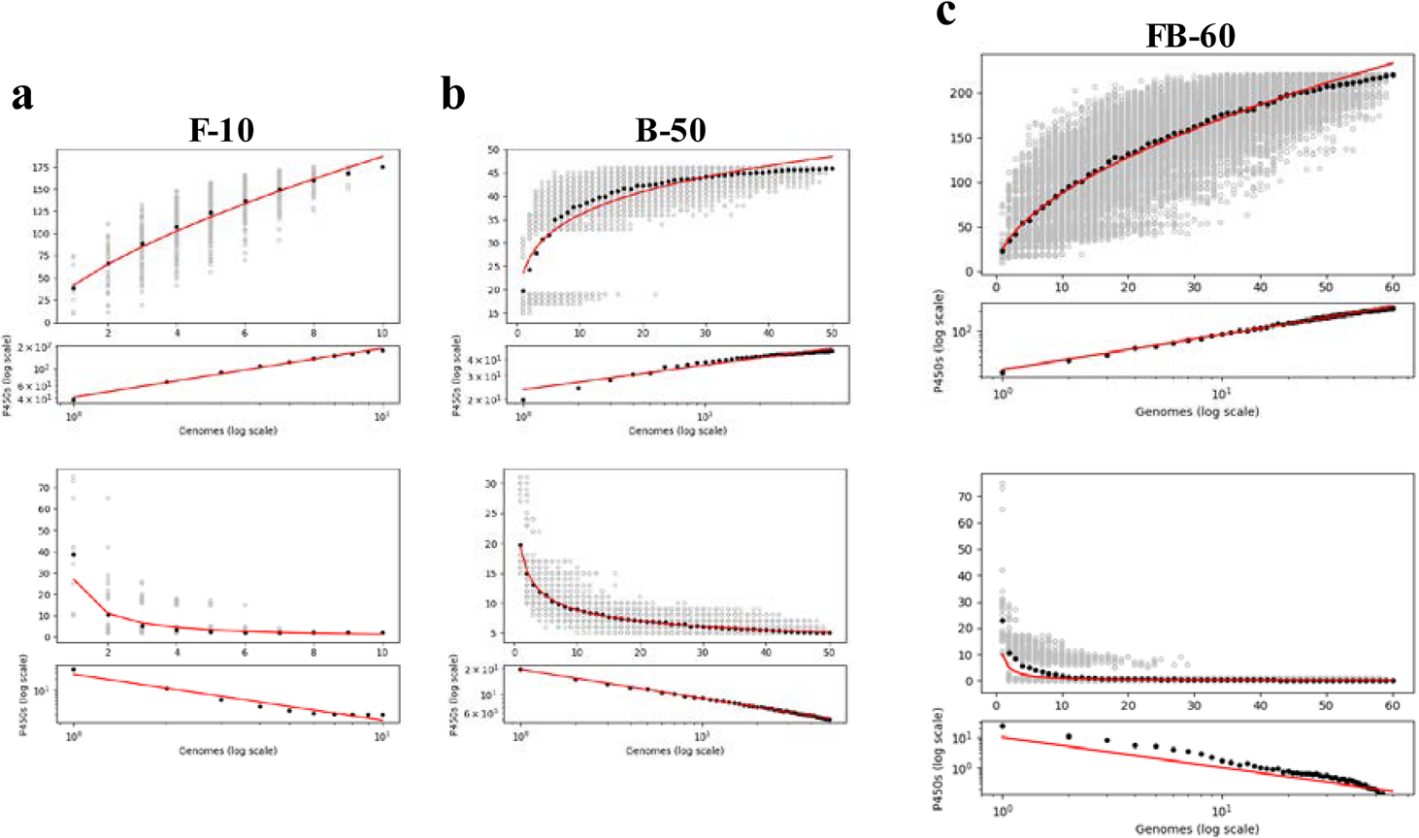
Pan-CYPome analyses. The pan-CYPome (core- and pan-CYPome) profile trends of the three datasets: (**a**), F-10; (**b**), B-50; (**c**), FB-60. The open and closed circles indicate the number of CYPs of the pan-CYPome and core-CYPome of *n* genomes and their average pan-CYPome size and core CYPome size, respectively. The red curves represent a power law fitting of the data. Please refer text for a detailed description of the pan-CYPome figures. The datasets and their results, including high-quality images, can be downloaded from https://github.com/Okweon/CYPminer

From 117,916 protein sequences of the 10 fungal genomes (sample F-10), CYPminer initially generated 71,023 clusters (Table 1). Using these representative sequences (i.e., 71,023 centroids), CYPminer identified 618 CYPs, classified into 175 families, including 25 new families and 307 subfamilies, including 169 new subfamilies. Analysis of CYP families revealed that the CYP65 (30 members), CYP509 (19 members), and CYP203 (19 members) were the dominant CYP families (Table 1). Pan-CYPome analysis of the F-10 found two core CYP families, CYP51 and CYP61 (Fig. 3a), which consist of house-keeping CYPs found in almost all fungi, plants and animals [10], and two core subfamilies CYP51F and CYP61A in the 10 fungal genomes, respectively. The size of CYPome of individual strains of sample F-10 is consistent with reference data such as the Fungal Cytochrome P450 Database (FCPD) (http://p450.riceblast.snu.ac.kr/index.php?a=view), strongly supporting its functional quality.

In the case of sample B-50 (230,489 sequences and 17,623 clusters), CYPminer identified 1286 CYPs, classified into 46 families, including 5 new families, and 65 subfamilies, including 14 new subfamilies (Table 1). We also note that a new family (with 31.7% identity to CYP141A1) was the dominant family with 84 members, followed by CYP135 with 81 members and CYP140 with 61 members (Table 1). Five CYP families (CYP124, CYP136, CYP138, CYP140, and CYP144) were conserved in the 50 mycobacterial genomes (Fig. 3b). The size of the CYPome of sample B-50 matches well with those of other mycobacterial reference data [6].

From 60 genomes consisting of 10 fungal and 50 mycobacterial genomes (348,405 sequences and 88,648 clusters) of sample FB-60, CYPminer identified and classified a total of 1904 CYPs: 220 CYP families (30 new families), including 175 CYPs from fungal genomes and 46 CYPs from mycobacterial genomes, and 372 subfamilies (183 new subfamilies), including 307 fungal CYP subfamilies and 65 bacterial subfamilies (Table 1). As a result, CYP51 is the only CYP family present in both kingdom genomes and is one of the most dominant families with 64 members (14 from 10 fungal genomes and 50 from 49 mycobacterial genomes). However, the CYP51s belong to two different subfamily groups: CYP51F for fungi and CYP51B for mycobacteria. No core CYP was observed in the sample FB-60 (Fig. 3c). Overall, the output data of sample FB-60 satisfy the equation *C*_*FB-60*_ = C_F*-10*_ + *C*_*B-50*_ - (C_F*-10*_ ∩ *C*_*B-50*_), where *C*_*x*_ is the CYP number found from sample x, from the three sample datasets. This result clearly supports the functionality and utility of CYPminer across kingdoms.

**Fig. 4:**
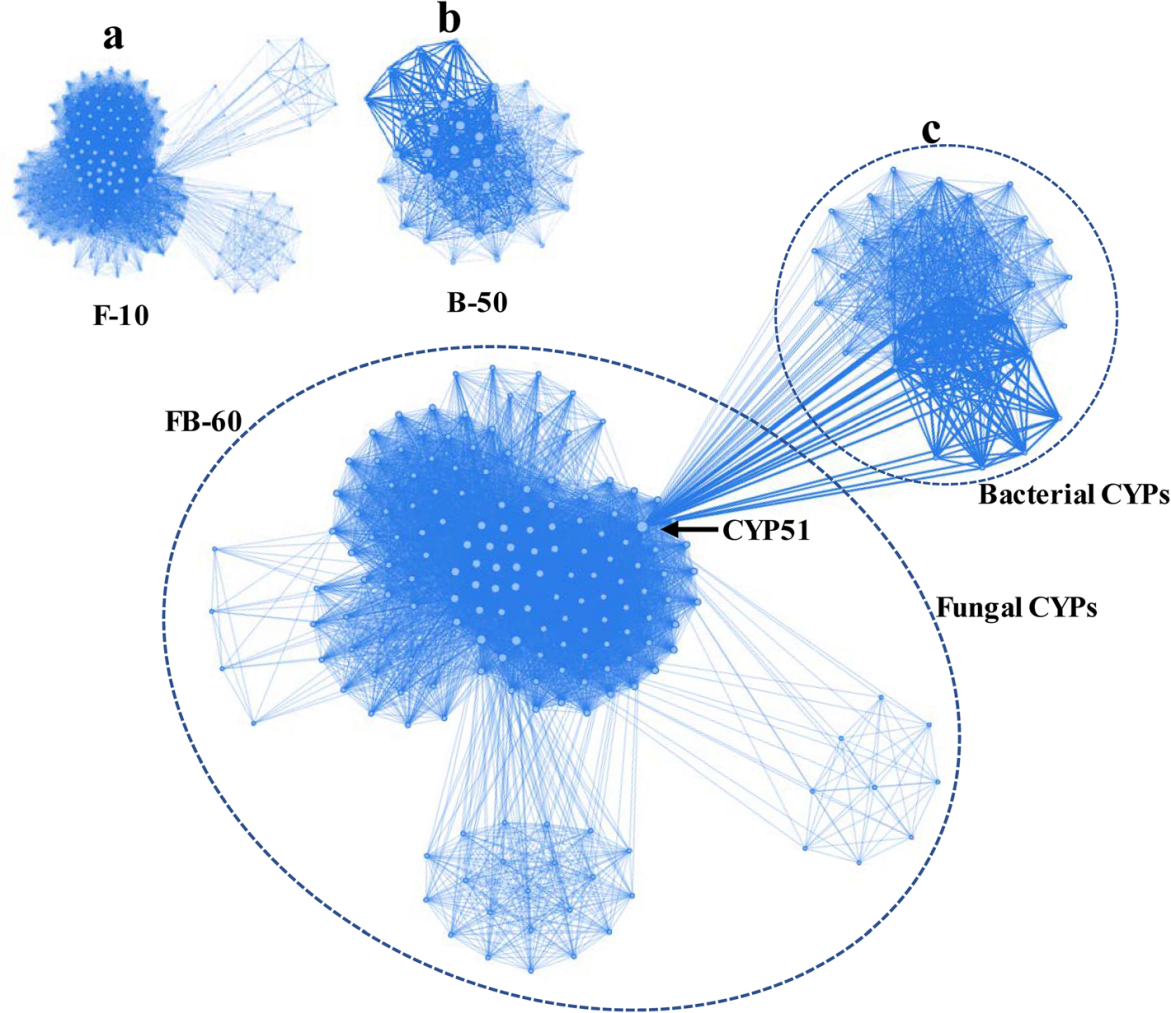
Co-occurrence network analyses. **a**, **b**, and **c** represent the CYP family co-occurrence networks for samples F-10, B-50, and FB-60, respectively

**Fig. 5:**
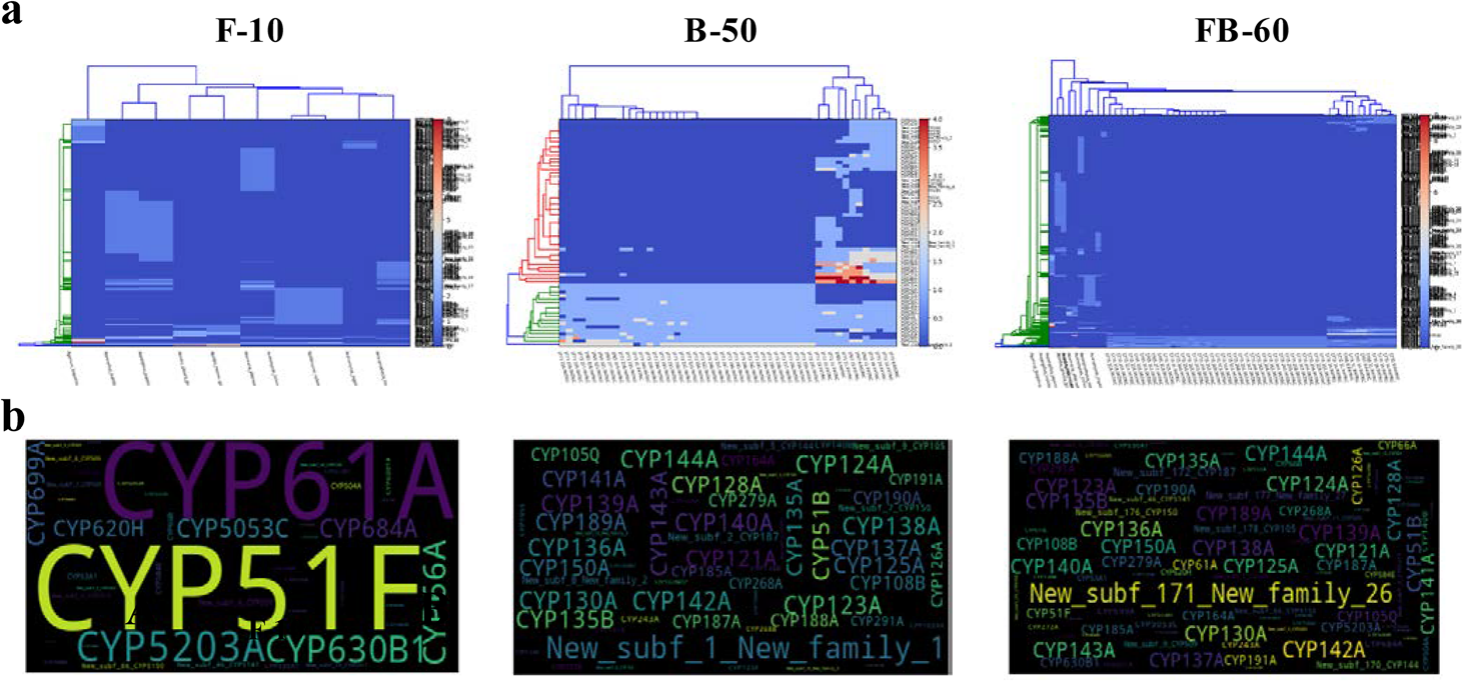
CYP subfamily-based genome clustering (**a**) and CYP clouds (**b**) of the three datasets (F-10, B-50, and FB-60). The datasets and their results, including high-quality images, can be downloaded from https://github.com/Okweon/CYPminer

A set of graphical outputs of CYPminer help to understand the degree of diversity and dynamics of CYPs in a genome population. Figure 3 shows pan-CYPomic data of the three samples. The pan-CYPome of sample F-10 shows ‘open’ pan- and ‘closed’ core-CYPomic properties, i.e., dramatically increasing the size of the pan-CYPome but not changing the size of the core-CYPome by adding new genomes (Fig. 3a). In case of sample B-50, both pan- and core-CYPome are almost ‘closed’ forms (Fig. 3b). However, the addition of 10 fungal genomes (F-10) to the 50 mycobacterial population (B-50) had an apparent impact on the pan-CYPome (FB-60): rapidly increasing the ‘open’ pan-CYPome by addition of new genomes but the completely ‘closed’ core-CYPome after ~ 20 population size (Fig. 3c). Such pan-CYPomic changes of the FB-60 indicates significantly different CYP profiles of the two kingdoms, as revealed in the identification/classification data. The other graphical data [i.e., CYP co-occurrence networks (Fig. 4), CYP-centric genome clustering (Fig. 5a), and CYP clouds (Fig. 5b)] further support the pan-CYPomic observation. As shown in Fig. 5a, a clustered heatmap with dendrograms, directly visualized the GCMs without the need for dimensionality reduction, provides insights into CYP-centric genome-wide association. In the F-10 clustered heatmap, the 10 fungal genomes can be clustered into a few clusters with the outliers fused in rather arbitrarily at much higher distances (Fig. 5 [F-10]). The CYP-centric clustering pattern of the 10 fungal genomes (sample F-10) explains well the linear shape of the ‘open’ F-10 pan-CYPome (Fig. 3a). On the other hand, the 50 mycobacterial genomes can be grouped into several clusters with a few outliers (Fig. 5 [F-10]). Interestingly, about 38 mycobacterial genomes can be grouped into a cluster with similar CYP profiles and in this case, there are only 3 outliers. Such clustering pattern of the 50 mycobacterial genomes supports the sigmoidal shape (or sharpness) of the F-50 pan-CYPome which is almost ‘closed’ (or saturated) form (Fig. 3b). In this respect, the degree of clustering of the FB-60 (Fig. 5 [FB-60]) agrees with the sharpness of FB-60 pan-CYPome (Fig. 3c). The CYP clouds also graphically support the degree of frequency and diversity of CYPs in a genomic population (Fig. 5b). Conclusively, CYPminer generates a set of graphical data with mutually supportive relationships in terms of the degree of diversity and dynamics of CYPs in a genome papulation.

The CYP co-occurrence network provides a graphic visualization of the collective interconnection of CYPs based on their paired presence within a genome. All the CYP co-occurrence networks are scale-free with apparent connection preferences (Fig. 4). As shown in the CYP family co-occurrence network of sample FB-60 (Fig. 4c), the two networks of sample F-10 (Fig. 4a) and sample B-50 (Fig. 4b) are connected via CYP51, one of the hub nodes with a relatively high connection degree. In the three networks, CYP51 has hub node properties that consist of a relatively big node and a high connection degree with thick width, indicating its high frequency and co-occurrence in the genomes. On the other hand, in the subfamily co-occurrence network, the two networks of sample F-10 and sample B-50 are not connected due to the loss of the hub node CYP51 caused by its different subfamily classification (data not shown).

CYPminer provides a set of graphical data which have unique graphical information but mutually supportive relationships. Together with the tables, systematic integration and interpretation of the graphical outputs are essential to understand the real CYP world, which has its own structural, behavioral, and evolutionary features.

## Conclusion

In this report, we present an automated computational pipeline for identification, classification, and downstream analyses of CYPs at the genome level. We demonstrate that our CYPminer is robust enough for large-scale CYP analyses from all kingdoms with a user-friendly graphic interface, essential for systematic genome annotation and comparative insights in terms of CYPs. CYPminer also can be extended and adapted easily for broader usage.

## Availability of data and materials

**Project name:** CYPminer.

**Project home page:** https://github.com/Okweon/CYPminer

**Operating system(s):** Windows.

**Programming language:** Python 2.7.

**License:** GPL v3.

**Any restriction to use by non-academics:** not applicable.

## Data Availability

An executable file of CYPminer, the related databases, and test datasets are available at https://github.com/Okweon/CYPminer.

## Abbreviations

CYP: Cytochrome P450s
GCM: Genome-CYP Matrix
